# Serum levels of endocannabinoids and related lipids in painful vs painless diabetic neuropathy: results from the Pain in Neuropathy Study

**DOI:** 10.1097/j.pain.0000000000003015

**Published:** 2023-08-11

**Authors:** Emmanuel Bäckryd, Andreas Themistocleous, Niclas Stensson, Andrew S. C. Rice, Solomon Tesfaye, David L. Bennett, Björn Gerdle, Bijar Ghafouri

**Affiliations:** a Pain and Rehabilitation Center, and Department of Health, Medicine and Caring Sciences, Linköping University, Linköping, Sweden; b Nuffield Department of Clinical Neurosciences, University of Oxford, Oxford, UK; c Pain Research, Department Surgery and Cancer, Faculty of Medicine, Imperial College London, United Kingdom; d Diabetes Research Unit, Sheffield Teaching Hospitals NHS Foundation Trust, Sheffield, United Kingdom

**Keywords:** Biomarker, Chronic pain, Diabetes, Endocannabinoids, *N*-acylethanolamines, Neuropathic, Neuropathy, Pain, Polyneuropathy

## Abstract

N-arachidonoylethanolamine (also known as anandamide) and 2-arachidonoylglycerol are activators of the cannabinoid receptors. The endocannabinoid system also includes structurally and functionally related lipid mediators that do not target cannabinoid receptors, such as oleoylethanolamide, palmitoylethanolamide, and stearoylethanolamide. These bioactive lipids are involved in various physiological processes, including regulation of pain. The primary aim of the study was to analyze associations between serum levels of these lipids and pain in participants in the Pain in Neuropathy Study, an observational, cross-sectional, multicentre, research project in which diabetic patients with painless or painful neuropathy underwent deep phenotyping. Our hypothesis was that painful neuropathy would be associated with higher levels of the 5 lipids compared with painless neuropathy. Secondary aims were to analyze other patient-reported outcome measures and clinical data in relationship to lipid levels. The lipid mediators were analyzed in serum samples using liquid chromatography tandem mass spectrometry (LC-MS/MS). Serum levels of anandamide were significantly higher in the painful group, but the effect size was small (Cohen d = 0.31). Using cluster analysis of lipid data, patients were dichotomized into a “high-level” endocannabinoid group and a “low-level” group. In the high-level group, 61% of patients had painful neuropathy, compared with 45% in the low-level group (*P* = 0.039). This work is of a correlative nature only, and the relevance of these findings to the search for analgesics targeting the endocannabinoid system needs to be determined in future studies.

## Abstract 2

Supplemental Digital Content is Available in the Text.

Painful diabetic neuropathy was associated with high serum levels of endocannabinoids and related lipids in general, and with *N-*arachidonoylethanolamine (anandamide) in particular.

## 1. Introduction

Endocannabinoids (eCBs) are a heterogenous group of lipid messengers, which activate the cannabinoid receptors 1 and 2 (CB1, CB2). CB1 is widely distributed through the nervous system and particularly enriched in nociceptive pathways, whereas CB2 shows higher expression in the immune system, including microglia.^18,20,33^ The 2 best known ligands are *N-*arachidonoylethanolamine (AEA, also known as anandamide) and 2-arachidonoylglycerol (2-AG). The eCB system also includes lipid mediators that are structurally and functionally related to eCBs but do not target CB receptors, namely, *N*-acylethanolamines such as oleoylethanolamide (OEA), palmitoylethanolamide (PEA), and stearoylethanolamide (SEA).^3,14,22,36,46^

Studies in preclinical animal models suggest that cannabinoids and modulators of endocannabinoids might be effective analgesics,^18,34,61^ cannabinoids having antinociceptive effects and anti-inflammatory properties, for example, through modulation of synaptic transmission, induction of apoptosis, inhibition of cell proliferation, suppression of cytokine production, and induction of T-regulatory cells.^16,18,38,51^

Despite encouraging preclinical literature from animal experiments, cannabis-based medicines have not been shown to be effective and safe for the treatment of (chronic) pain in everyday clinical practice.^19,57^ Hence, there is a translational gap between preclinical and clinical studies. A research agenda has recently been published by a task force from the *International Association for the Study of Pain*, and one of the main basic and translational research priorities was “to elucidate the neurobiology of endocannabinoid signaling in relation to pathological pain processing”.^30^ We have previously reported that blood levels of 2-AG, OEA, PEA and SEA, but not AEA, were elevated in female patients with fibromyalgia compared with healthy control subjects^55^; moreover, a positive, multivariate, intercorrelation pattern was described by principal component analysis between OEA, PEA, SEA, and AEA. In addition, women with chronic widespread pain had higher plasma levels of OEA and PEA compared with healthy control subjects,^54^ and in fibromyalgia patients, exercise seems to increase anandamide.^53^

The pathophysiological mechanisms underlying the development of pain in patients with diabetic distal symmetric polyneuropathy (DSP) are poorly understood.^12,39,43,48–50^ Risk factors for the development of painful DSP include obesity, glycemic burden, and female sex.^45^ We have recently shown that low-grade systemic inflammation (and HGF, CSF-1, and CD40 in particular) was related to the severity of neuropathy and neuropathic pain in a subgroup of patients with diabetic DSP recruited in the Pain in Neuropathy Study (PiNS).^12,58^ There is a potential physiological link between inflammation and the eCB system.^16,38^

The primary aim of the present biomarker^4^ study was to analyze associations between eCBs (2-AG, AEA) and related lipids (SEA, OEA, PEA) measured in serum and pain in PiNS participants. Based on previous findings,^55^ our hypothesis was that painful DSP would be associated with higher levels of eCBs and related lipids in serum, compared with painless DSP. Secondary aims were to analyze other patient-reported outcome measures and clinical data in relationship to lipid levels.

## 2. Methods

### 2.1. Participants and clinical data

In this study, we included a total of 170 participants from PiNS, which is an observational, cross-sectional, multicentre study in which patients with painless or painful DSP underwent deep phenotyping.^58^ Participants were included consecutively in PiNS until target number was reached. Participants included in this biomarker study were those for whom serum and neuropathic pain grading according to IASP/NeuPSIG were available.^59^ We applied the NeupSIG grading system for neuropathic pain to assess pain in the feet as being the plausible anatomical distribution when separating those with painful vs painless diabetic neuropathy.

Pain in Neuropathy Study inclusion criteria were diabetes mellitus in patients aged 18 years and older with diagnosed DSP or with symptoms and signs suggestive of DSP. Exclusion criteria were pregnancy, coincident major psychiatric disorders, poor or no English language skills, severe pain at recruitment from a cause other than DSP (to prevent potential confounding influence on pain reporting and psychological and quality-of-life–reported outcomes), patients with documented central nervous system lesions, or patients with insufficient mental capacity to provide informed consent or to complete questionnaires. Many of the study participants were recruited from primary care practices in London and Oxford. Study participants were also recruited from diabetes and other clinics at Chelsea and Westminster Hospital (London), Sheffield Teaching Hospitals and Oxford University Teaching Hospitals, neurology clinics at King's College Hospital (London), and through advertisements.

The following clinical data were analyzed in this study:

(1) Anthropometric data (weight (kg) and height (m))
(2) Data pertaining to diabetes and metabolic control (body mass index (BMI; kg/m^2^), HbA1c, type of diabetes, duration of diabetes)
(3) Data related to neuropathy—the Toronto Clinical Scoring System (TCSS) correlates with diabetic neuropathy severity and was used as a screening tool for DSP^6^
(4) Neuropathic pain questionnaires: the Douleur Neuropathique en 4 Questions (DN4)^5^; PainDETECT^21^
(5) Intraepidermal Nerve Fibre Density (IENFD)
(6) Pain intensity (pain diary mean of numerical rating scale [NRS, 0 indicating no pain and 10 worst pain imaginable] for 7 days) for patients with painful neuropathy; mild pain defined as NRS < 4.0, moderate pain as NRS 4.0 to 6.9, and severe pain as NRS ≥7.0^32^
(7) Questionnaires pertaining to psychological distress: the Depression Anxiety Positive Outlook instrument (DAPOS) was used to measure mood and anxiety by 3 subscales (DAPOS anxiety, DAPOS depression, DAPOS positive outlook)^42^ and the Pain Anxiety Symptom Scale 20 (PASS) to assess pain-related anxiety^37^
(8) Pain catastrophizing scale (PCS) was used to assess the cognitive process by which pain was appraised.^41^ Catastrophizing is characterized by a lack of confidence and control and an expectation of negative outcomes.
(9) Insomnia severity index (ISI)^2^

The methods and questionnaires have been previously described in detail.^58^ Information on ethnicity was not available.

### 2.2. Blood sampling

A 10-mL nonfasting blood sample (BD Vacutainer SST Tubes) was drawn from each participant. Blood samples were collected at around either 12:00 or 16:00 (±30 minutes). Participants were seen at either 09:00 or 13:00, and samples were taken at the end of the visit once all other procedures were completed. After 30 minutes, to allow blood to clot, the sample was centrifuged at 1784 g for 10 minutes at a temperature of 4°C. Serum was then aliquoted into 1.8-mL Nunc CryoTubes and immediately stored at −80°C. Hence, samples were placed in freezer within 45 minutes of the blood draw (30 minutes for blood to clot, 10 minutes to centrifuge, and 5 minutes to aliquot).

### 2.3. Analysis of lipid concentrations

The lipid mediators were analyzed in serum samples using liquid chromatography tandem mass spectrometry (LC-MS/MS) based on a previously published method.^56^ Briefly, the serum samples were thawed, and a mixture of internal standards containing 50 nM of AEA-d4, OEA-d4, PEA-d4, and SEA-d3 and 1 µM of 2AG-d5 (Cayman Chemicals) was added to the samples, and the lipids were extracted using C8 solid phase columns (Biotage, Uppsala, Sweden) as described previously.^1^ The extracted lipids were analyzed using liquid chromatography coupled to a Quantum triple quadrupole mass spectrometer equipped with an electrospray ionization source (TSQ Quantum Access, Thermo Scientific) as described previously.^55^ Selected reaction monitoring (SRM) was used for the targeted lipid analysis using following MS/MS transitions: m/z 348.3/62.4, 326.3/62.4, 300.3/62.4, 328.3/62.4, and 379.3/287.3 for AEA, OEA, PEA, SEA, and 2-AG. The obtained raw data from mass spectrometry analysis were processed using Xcalibur (version 2.1, Thermo Scientific) software. Quantification of the analytes was performed using isotopic dilution according to the area ratio of their corresponding deuterated internal standard signal area. The linearity of the measuring ranges was assessed with standard curves using 6 points ranging from 1 to 25 nM for AEA and from 10 to 500 nM for OEA, PEA, and SEA, and from 50 to 1250 nM for 2-AG in duplicate. Linear regression and χ^2^ weighting were applied.

### 2.4. Statistics

Details of multivariate data analysis (MVDA) methodology^17,62^ have been described in previous publications.^7–11,26,27,40^ Briefly, we used SIMCA version 16 (Sartorius Stedim Biotech, Umeå, Sweden) for MVDA computations on endocannabinoid data. We performed principal component analysis (PCA), orthogonal partial least squares regression of pain intensity (OPLS), and hierarchical clustering analysis (HCA). Principal component analysis is a technique that models the correlation structure of a data set and can be used for multivariate correlation analysis and for the identification of multivariate outliers.^17,62^ Principal components (PC) extract relevant information found in the data, reducing a high-dimensional space (high number of variables) to a few “summary variables.” After outlier detection with PCA (strong outliers defined as Hotelling T2>>T2Crit(99%) and moderate outliers as DModX>2*DCrit), we regressed pain intensity (the Y variable) by OPLS using lipid data as X variables. The importance of the X variables for the OPLS model was quantified as a variable influence on projection value (VIP or VIPpred if more than one component is identified). VIP (and VIPpred) indicates the relevance of each X-variable pooled over all dimensions and Y variables—that is, VIP (and VIPpred) values indicate the variables that best explain Y. Variables with a VIP or VIPpred >1.0 and having a 95% confidence interval not including zero are usually considered significant. We also applied a bottom–up HCA to the principal component score vectors using the default Ward linkage criterion to identify relevant subgroups of patients. Hierarchical clustering analysis can be viewed as a complement to PCA in the sense that although PCA identifies distinct clusters in multivariate space, HCA can find subtle clusters. In addition, PCA-defined clusters are determined (if at all possible) subjectively by the researcher based eg, on an inspection of the score plot, whereas HCA generates a dendrogram where group belonging is defined statistically by SIMCA. Hence, in the resulting dendrogram of our HCA, we identified clusters (=subgroups), and clinical data were the compared between the subgroups to ascertain the clinical relevance of the subgroups.

In the text and tables, data are presented as median (interquartile range, IQR), if not otherwise specified. For computations with Kruskal–Wallis test, Mann–Whitney *U* test, χ^2^ test, and Spearman rho for bivariate correlations, we used IBM SPSS Statistics (version 24.0; IBM Corporation, Route 100 Somers, New York). A significance level of 0.05 was chosen. To handle the multiple testing problem, a false discovery rate (FDR) at the 20% level was applied using the Benjamini–Hochberg procedure instead of Bonferroni corrections.^28^

### 2.5. Ethics

The study was approved by the National Research Ethics Service of the United Kingdom (No.:10/H07056/35). All study participants signed written consent before participating.

## 3. Results

### 3.1. Painless vs painful distal symmetric polyneuropathy

Clinical data in painless vs painful neuropathy are shown in Table 1. All patients in the painful neuropathy group had *definite* neuropathic pain (except one who had probable neuropathic pain), reported a pain diary NRS pain intensity of 4.9 (2.8-6.8), and had a pain duration as follows: 4% had been in pain 3 to 12 months, 44% reported 1 to 5 years, and 52% reported >5 years.

**Table 1:** Clinical data in patients with painless vs painful neuropathy.

	Painless neuropathy N = 79	Painful neuropathy N = 91	*P*
Age (y)	69 (62-74)	65 (57-72)	0.088
Sex (% females)	27%	29%	0.772
HbA1c (%)	7.3 (6.6-8.0)	7.8 (6.9-9.1)	0.005*
Body mass index (BMI; kg/m^2^)	29.6 (25.7-33.5)	31.0 (27.2-36.4)	0.193
Duration of diabetes (y)	14.2 (9.8-22.2)	13.7 (7.9-21.0)	0.227
Type 2 diabetes (%)	94%	90%	0.400
IENFD	1.2 (0.4-1.8)	0.8 (0.2-1.6)	0.335
TCSS total score	8 (5-10)	12 (9-14)	<0.001*
PainDETECT	2 (0-6)	16 (11-21)	<0.001*
DN4	2 (1-3)	6 (4-7)	<0.001*
PASS total	7 (0-16)	22 (9-44)	<0.001*
DAPOS anxiety	3 (3-4)	4.5 (3-7)	0.002*
DAPOS depression	6 (5-8)	7 (5-11)	0.020*
DAPOS positive outlook	12 (10-14)	10.5 (9-13)	0.030*
PCS total	6.5 (0-14)	14.0 (7-26)	<0.001*
ISI total score	6.5 (1.3-11.0)	13.0 (6.0-19.8)	<0.001*

Median (interquartile range) if not percentage. Furthest to the right is the result of the statistical comparisons between the 2 groups (*P* values).

* Statistically significant at the 0.05 level.

Serum levels of AEA were higher in the painful group (*P* = 0.047, Table 2). This finding remained statistically significant when applying a false discovery rate of 20% according to the Benjamini–Hochberg procedure, but the effect size was small (Cohen d = 0.31). Results with the painful neuropathy group stratified into clinically relevant categories, that is, mild, moderate, and severe pain intensities, are shown in Online supplement 1 (available at http://links.lww.com/PAIN/B896).

**Table 2:** Serum levels of endocannabinoids and related lipids in painless vs painful neuropathy.

Substance	Painless neuropathy N = 79	Painful neuropathy N = 91	*P*
2-AG (nM)	55.5 (36.7-79.9)	68.7 (46.8-111.6)	0.100
AEA (nM)	0.93 (0.57-1.43)	1.07 (0.71-1.62)	0.047*
OEA (nM)	7.61 (5.45-10.16)	8.20 (6.11-11.04)	0.154
PEA (nM)	6.12 (5.12-7.84)	6.75 (5.47-8.79)	0.084
SEA (nM)	6.00 (4.68-6.93)	5.61 (4.56-8.05)	0.736

Median (interquartile range). Furthest to the right is the result of the statistical comparisons between the 2 groups (*P* values).

* Statistically significant at the 0.05 level; this remained significant at FDR of 20%.

### 3.2. Multivariate intercorrelations between the lipids

**Figure 1.**
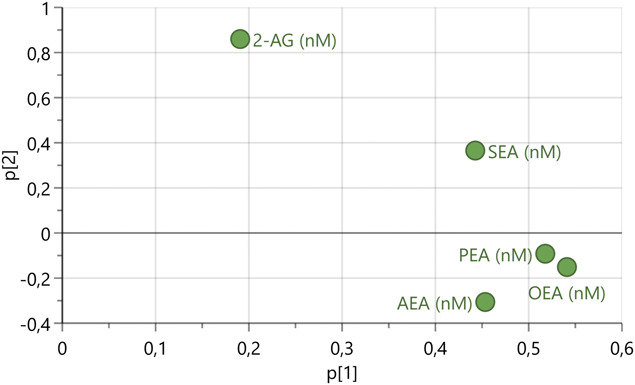
Loadings plot of principal components (PC) analysis model of 5 endocannabinoids and related lipids. N = 170, 2 PC, R^2^ = 0.75, Q^2^ = 0.25. The 2 axes represent the loadings of the 2 PC. Note that only the first PC p[1] was significant.

A PCA was computed on the 5 studied lipids (n = 170, 1 PC, R^2^ = 0.56, Q^2^ = 0.31). No outlier was found. To better illustrate the multivariate pattern, an extra PC was added to the model, hence generating av loadings plot with 2 axes (Fig. 1). As seen in Figure 1, the strongest intercorrelation was found between PEA and OEA (shortest distance to each other in the plot), and 2-AG was the least correlated to the other lipids (and was more strongly loaded upon the second nonsignificant component). This overall pattern was confirmed by bivariate correlations, see correlation matrix in Online supplement 2 (available at http://links.lww.com/PAIN/B896).

### 3.3. Clustering of patients according to lipid profile and comparison of clinical data between the groups

Next, HCA was done using lipid data, and 2 subgroups were defined, here labelled clusters A and B. Cluster B had significantly higher levels of all 5 eCBs/related lipids and was thus a “high-eCB group,” whereas cluster A was a “low-eCB group” (Table 3). In cluster A (n = 76), 45% of patients had painful neuropathy, compared with 61% of patients in cluster B (n = 94) (*P* = 0.039) (Table 4). Moreover, cluster B had higher levels of DAPOS depression compared with cluster A (*P* = 0.04), but this was no longer significant at FDR of 20% (Table 4). Other patient-reported measures did not differ between the clusters, and neither did age, sex, or diabetes-related data (Table 4).

**Table 3:** Levels of eCBs and related lipids in the 2 clusters defined by hierarchical cluster analysis (HCA).

	Cluster A, “low eCB” N = 76	Cluster B, “high eCB” N = 94	*P*
2-AG (nM)	59.03 (42.30-72.80)	76.56 (42.07-131.63)	0.010
AEA (nM)	0.69 (0.50-0.92)	1.42 (1.01-1.78)	<0.001
OEA (nM)	5.78 (5.05-7.05)	10.19 (8.37-12.79)	<0.001
PEA (nM)	5.29 (4.58-5.79)	8.09 (6.80-9.56)	<0.001
SEA (nM)	4.72 (3.90-5.49)	6.84 (5.83-8.72)	<0.001

Median (interquartile range). Furthest to the right is the result of the statistical comparisons between the 2 groups (*P* values).

**Table 4:** Frequency of painful neuropathy and other clinical data in 2 clusters of patients defined by hierarchical cluster analysis (HCA).

	Cluster A, “low eCB” N = 76	Cluster B, “high eCB” N = 94	*P*
Primary aim analysis			
Painful neuropathy (%)	45%	61%	0.039*
Secondary aim analyses			
Age (y)	66 (60-74)	69 (59-73)	0.965
Sex (% females)	21%	33%	0.084
HbA1c (expressed in %)	7.55 (6.73-8.48)	7.50 (6.70-8.46)	0.966
Body mass index (BMI; kg/m^2^)	29.7 (25.9-33.4)	30.1 (27.4-36.3)	0.115
Duration of diabetes (y)	14.3 (9.5-20.9)	13.4 (8.3-21.8)	0.440
Type 2 diabetes	91%	93%	0.677
IENFD	1.1 (0-1.7)	0.9 (0.4-1.7)	0.817
TCSS total score	10 (7-12)	10 (7-13)	0.813
PainDETECT	8 (2-18)	11 (2-17)	0.705
DN4	3 (2-6)	4 (2-6)	0.396
PASS total	14 (4-31)	14 (6-33)	0.656
DAPOS anxiety	3 (3.0-5.3)	4 (3-7)	0.135
DAPOS depression	6 (5-8)	7 (5-12)	0.040*
DAPOS positive outlook	11 (9-13)	9 (11-14)	0.514
PCS Total	9 (4-20)	11 (3-24)	0.449
ISI Total score	8.5 (4.0-14.0)	10.0 (4.8-16.3)	0.353

Data in the table expressed as median (interquartile range), if not %. Furthest to the right is the result of the statistical comparisons between the 2 groups (*P* values).

* Statistically significant at the 0.05 level. For the 16 secondary aim analyses listed in the table, FDR of 20% was applied, whereby the difference in DAPOS depression was no longer statistically significant (the *P* value of 0.040 being higher than the corresponding critical value).

**Figure 2.**
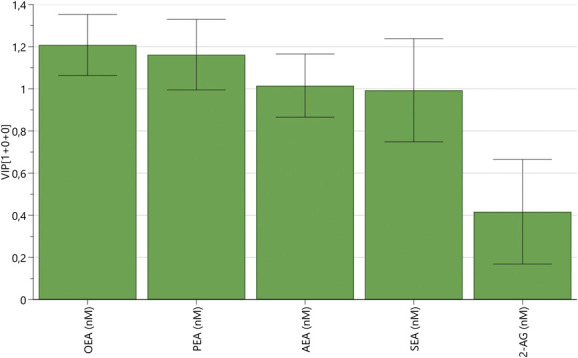
Variable influence on projection (VIP) values with 95% confidence interval in decreasing order of magnitude in OPLS-DA model exploring the relative importance of each substance for cluster discrimination (cluster A vs cluster B).

An OPLS-DA model was computed to explore the relative importance of each of the 5 lipids for cluster membership discrimination (ie, belonging to cluster A or B). The model had n = 170, 1 latent variable (the predictive one), R^2^ = 0.66 and Q^2^ = 0.65, *P* < 0.001 by CV-ANOVA. We found that OEA was the most important lipid for group discrimination (VIP = 1.21), followed by (in descending order of VIP) PEA (VIP = 1.16), AEA (VIP = 1.02), SEA (VIP = 0.99), and 2-AG (VIP = 0.42). Hence, 3 lipids had VIP > 1.0 (OEA, PEA and AEA), and the least important lipid for group discrimination was 2-AG (Fig. 2).

### 3.4. Investigation of potential confounders

Finally, to investigate the potential confounding effect of the clinical data listed in Table 1, we used the score t1 of the PCA model described above (n = 170, 1 PC, R^2^ = 0.56, Q^2^ = 0.31) as a summary measure for the 5 studied lipids. The score t1 was used as Y variable, and the clinical data listed in Table 1 as X variables in 3 OPLS models as follows. For patients with painful DSP (n = 79), it was not possible to build a model at all. For patients with painless DSP (n = 91), a nonsignificant OPLS model was built (*P* = 1.0 by CV-ANOVA, 1 latent variable, R^2^ = 0.14, Q^2^=−0.08). For all patients taken together (n = 170), the OPLS model was also nonsignificant (*P* = 1.0 by CV-ANOVA, 1 latent variable, R^2^ = 0.08, Q^2^=−0.00). Hence, we found no evidence of a confounding effect of the variables listed in Table 1 on the levels of the 5 studied lipids taken as a whole.

## 4. Discussion

We have shown that high serum levels of 5 lipids belonging to the endocannabinoid system in general, and AEA in particular, were associated with painful as opposed to painless DSP. Although the effect size was small, the results are partially consistent with our original hypothesis that eCBs would be raised in painful vs painless DSP.

*N-*arachidonoylethanolamine is a partial agonist at cannabinoid receptors, the affinity for CB1 (predominantly expressed in the central nervous system) being 4 times higher than that for CB2 (mainly found on cells of the immune system).^52^ In chronic neurological disorders, altered levels of endocannabinoids such as AEA can conceptually be viewed either as a maladaptive mechanism contributing to disease or as an adaptive response aimed at restoring homeostasis.^15,33^ Viewed from that perspective, and because AEA is generally considered to be antinociceptive (eg, by activation of presynaptic CB1^52^), one could speculate that higher serum levels of AEA in painful diabetic neuropathy would mirror a compensatory mechanism, the organism trying to restore homeostasis in those more prone to developing a pain phenotype. Along the same interpretational lines, one could argue that the lower levels of AEA in severe neuropathic pain (see Online Supplement 1, available at http://links.lww.com/PAIN/B896) would be akin to having a less efficacious “endocannabinoid pain brake.” Although this is obviously very speculative, it seems to make sense physiologically. This would also mean that AEA would be more involved in compensatory mechanisms rather than in the generation of pain per se in diabetic neuropathy. Another way to put it is that, if pain is conceived as being the result of the balance between pronociceptive and antinociceptive factors, we have here only explored the antinociceptive side. From this perspective, this work can be viewed as an important complement to our previous article,^12^ in which we showed that belonging to a high-inflammation subgroup was associated with more neuropathy and higher pain intensity in patients with diabetic DSP. Hence, conceptually, one could speculate that our 2 articles together explore both the pronociceptive and antinociceptive side of painful diabetic DSP.

Different components of the endocannabinoid system are being investigated as potential targets for the development of new analgesics. CB1 and CB2 agonists are well-known in that respect. For instance, mixed CB1/CB2 agonism in a diabetic peripheral neuropathy model in rats led to reduced pain-related behavior.^60^ Generally speaking, however, exogenous cannabinoids targeting cannabinoid receptors have so far largely failed as analgesics in humans.^19,47,57^ The “drugability” of other components of the endocannabinoid system has therefore been investigated extensively. For instance, there are some indications that exogenous PEA could be used to treat pain,^24^ eg, because of its anti-inflammatory activity.^23^ Another major line of inquiry is about the inhibition of fatty acid amide hydrolase (FAAH) or monoacylglycerol lipase (MAGL), which are the principal enzymes responsible for the metabolism of AEA and 2-AG, respectively.^63^ Many FAAH inhibitors have been investigated in the pain context, eg, URB597, URB937, SA-57, PF-3845, and OL-135.^63^ FAAH inhibitors have generally been well tolerated by humans and lead to increased plasma AEA levels, but clinical trials have been rather disappointing in relation to analgesic efficacy.^20^ In addition, a trial with the FAAH inhibitor BIA 10-2474 revealed severe neurological adverse events, including one death, in 2016.^13,44^ Nonetheless, modulating the endocannabinoid system remains an active field of research,^20^ and animal experiments, eg, with FAAH knockout mice^35^ as well as case reports about pain insensitivity caused by a genetic loss-of-function disorder of FAAH,^29^ are powerful reminders of the potential effects of AEA and related lipids. One could speculate that for future AEA-enhancing medicines to be effective, it might be important to select patients with a combination of moderate-to-severe pain and low AEA levels, the rationale for this being that it would be those patients who would most likely benefit from enhancing the AEA “pain break.” To the best of our knowledge, this line of reasoning is a novel (albeit very speculative) way of relating AEA levels to subgroups based on pain intensity in patients with chronic pain.

The cluster analysis showed that a tendency to have higher levels of all the 5 lipids (ie, belonging to Cluster B) was associated with pain (Table 4). Once again, this could speculatively be interpreted as a compensatory mechanism in the organism's strive for homeostasis. Interestingly, of the 5 substances we measured, 2-AG was the one with the highest overlap of concentrations between the 2 clusters; this can be illustrated by the fact that it was the only substance for which the lower quartile value of cluster B was not higher than the higher quartile value of cluster A (Table 3). This is congruent with the fact that 2-AG was least correlated with other lipids (Fig. 1 and Online supplement 2, available at http://links.lww.com/PAIN/B896), thereby also replicating earlier findings from our group.^55^ This is not to say that we have shown 2-AG to be unimportant in this setting. If our study had had more power, perhaps, the tendencies apparent in Table 2 would have reached statistical significance? Such speculations notwithstanding, our OPLS-DA analysis between the clusters showed that high AEA was more distinctive for cluster B than high 2-AG (although OEA and PEA were more important than AEA). All in all, could it be that Fowler speculation^20^ that perhaps in humans, the pain regulatory response is a *combination* of effects (he mentions AEA and PEA) will turn out to be true?

A biomarker has been defined as “a characteristic that is objectively measured and evaluated as an indicator of normal biological processes, pathogenic processes, or pharmacologic responses to a therapeutic intervention.”^4^ There are limitations to this biomarker study. First, it has to be acknowledged that the effect sizes are small. However, the purpose of pain biomarker studies such as this one is not primarily to find a single clinically useful biomarkers highly discriminating between disease and nondisease. Rather, the purpose of this exploratory study is to help bridging the translational gap between animal experiments and clinical pain medicine by trying to mirror putative pathophysiological mechanisms present in humans (ie, “pathogenic processes” in the definition above). From that perspective, even minor effect sizes can be important to consider and report. The point is to ascertain if there is an endocannabinoid system “signal” to be found in painless vs painful diabetic neuropathy—and it seems that there is, not least concerning AEA. Hence, this study seems to support the notion that it is worthwhile to continue investigating the endocannabinoid system in neuropathic pain conditions.

Another limitation is the issue of possible confounders in this cross-sectional study. For instance, medication has not been taken into consideration. According to the literature, there is a positive correlation between plasma endocannabinoids and obesity.^25^ However, our investigation of possible confounders did not reveal any BMI effect (or any other effect of the clinical variables listed in Table 1). Moreover, eCB blood levels vary in relation to food intake^25^; in this study, the blood samples were not fasting samples. Whether this has influenced our findings or not is impossible to ascertain with any certainty. Also, there is a need for further investigations concerning the degree to which blood levels of eCBs relate to levels in the peripheral and central nervous system; can blood eCB concentrations really be used as biomarkers of eCB signaling status^31^? Being highly lipophilic, are they instead an indirect biomarker of general tissue eCB tone (eg, in muscle and adipose tissue)^31^?

Finally, we contend that one strength of this study is that we compared painful vs painless diabetic DSP and not painful diabetic DSP vs healthy control subjects. Given our interest in elucidating why some patients with neuropathy develop pain, whereas others do not, doing only the latter would have introduced a confounding effect of diabetes and neuropathy—making the interpretation of results even more difficult. By contrast, when investigating conditions where the pain is by definition the disease (eg, fibromyalgia), comparing with healthy control subjects is perfectly sensible (painless fibromyalgia being a contradiction in terms). Of course, the best option in this study would have been to have 3 groups instead of 2, that is, healthy control subjects vs painless neuropathy vs painful neuropathy—this was however not possible to achieve.

To conclude, in this correlative study, we have shown that in a cohort of patients with diabetic DSP, high levels of endocannabinoids and related lipids in general, and AEA (anandamide) in particular, were associated with painful neuropathy. The relevance of these findings to the search for analgesics targeting the endocannabinoid system needs to be determined in future studies.

## Conflict of interest statement

E.B., A.T.C., S.T., N.S., Bijar G., and Björn G have no conflict of interest to declare.

Andrew SC Rice declares interests occurring in the past 36 months.

(1) A.S.C.R. undertakes consultancy and advisory board work for Imperial College Consultants: in the past 36 months, this has included remunerated work for Confo, CombiGene, Vertex, Novartis, Orion, Shanghai SIMR Biotech & Science Practice (Wellcome Trust).

(2) A.S.C.R. is named as an inventor on patents: Rice A.S.C., Vandevoorde S. and Lambert D.M Methods using N-(2-propenyl)hexadecanamide and related amides to relieve pain. WO 2005/079771; Okuse K. et al. Methods of treating pain by the inhibition of vgf activity EP13702262.0/WO2013 110945.

(3) Grants and studentships: UKRI (Medical Research Council and BBSRC), Versus Arthritis, Alan and Sheila Diamond Trust, Royal British Legion, European Commission, Ministry of Defence, Dr Jennie Gwynn Bequests, The British Pain Society, and Royal Society of Medicine.

David Bennett has acted as a consultant in the past 2 years for Aditum Bio, Amgen, Biointervene, Bristows, LatigoBio, GSK, Ionis, Lexicon Pharmaceuticals, Lilly, Neuvati, Olipass, Regeneron, Replay, and TheraNexus on behalf of the Oxford University Innovation.

## Appendix A. Supplemental digital content

Supplemental digital content associated with this article can be found online at http://links.lww.com/PAIN/B896.

## Supplementary Material
