# Supplementary material for: Serum levels of endocannabinoids and related lipids in painful vs painless diabetic neuropathy: results from the Pain in Neuropathy Study
Source: Pain. 2023 Aug 11;165(1):225–32. doi: 10.1097/j.pain.0000000000003015 (PMC10723642; doi:10.1097/j.pain.0000000000003015)
Supplement: Supplementary file 1 [file jop-165-225-s001.pdf]

Online supplement 1

To further analyze pain intensity data, patients with painful neuropathy were stratified into mild pain, moderate pain and severe pain. Mild pain was defined as NRS 7 days mean pain < 4.0; moderate pain 4.0-6.9; severe pain ≥7. In the figures below, median values are represented by horizontal lines and the interquartile ranges (IQRs) by boxes. The ends of the whiskers represent minimum and maximum values, except when outliers and extremes are present. Outliers are defined as values above 3rd quartile + 1.5*interquartile range or below 1st quartile – 1.5*interquartile range. Extremes are defined as values above 3rd quartile + 3*interquartile range or below 1st quartile – 3*interquartile range. In the figures below, individuals above the whiskers are all outliers, except the following who are extremes: individuals 127 and 2 for AEA; 124 and 83 for 2-AG; 64 for OEA.

Omnibus testing was significant for AEA (Kruskal-Wallis H statistic=9.785, df=3, p=0.02) but not for the other lipids. Post hoc testing with Mann-Whitney U test (6 tests) showed that levels of AEA in the severe pain group were significantly lower than in the moderate pain group (p=0.021) and the moderate pain group had higher levels than the no pain group (p=0.003). These two differences remained significant at a false discovery rate of 20 % with computed critical values of 0.067 and 0.033, respectively. The four other post hoc tests were not significant; hence AEA levels did *not* differ between severe pain and no pain (p=0.82). The visual pattern of boxplots was similar for the other lipids except SEA, albeit not statistically significant, see below.

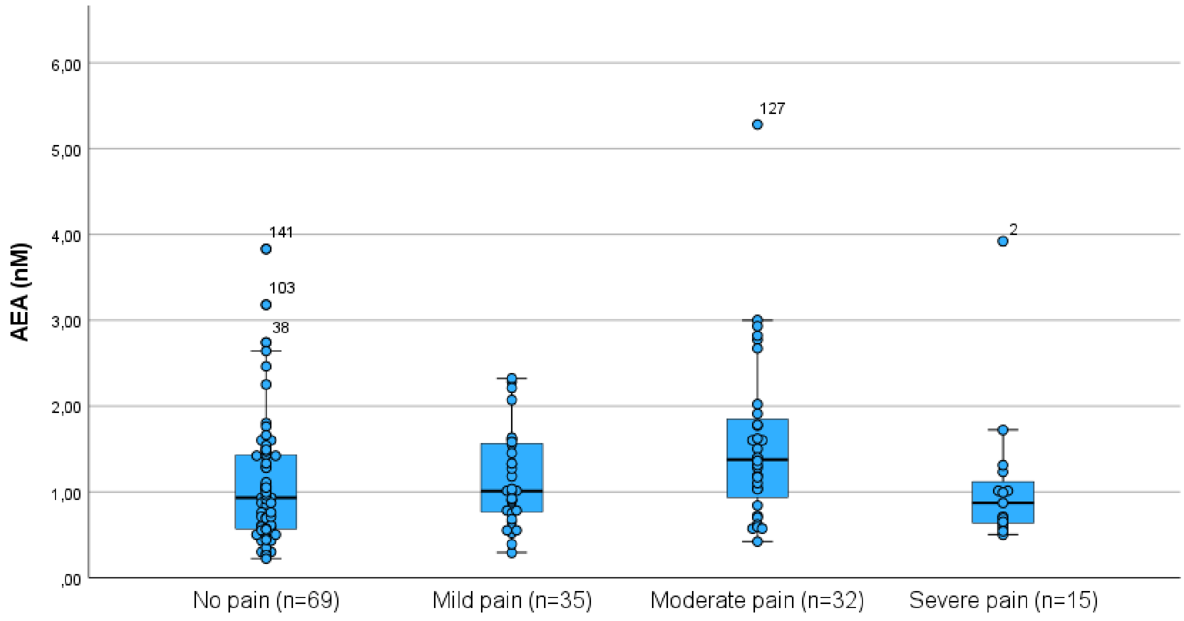

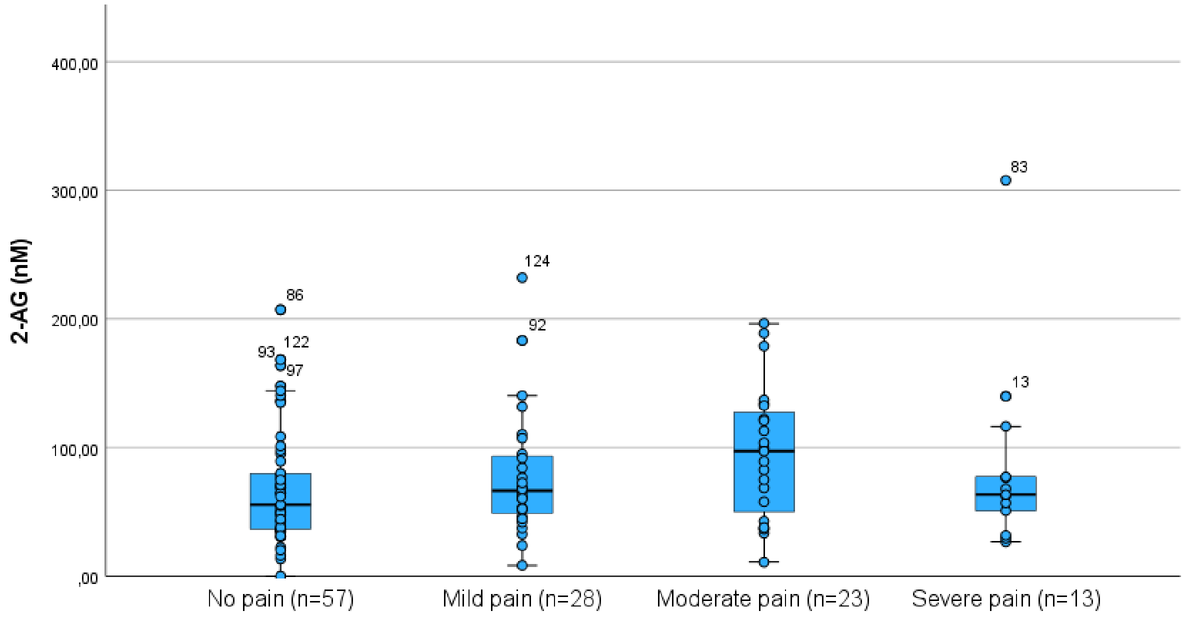
400,00
300,00
200,00
100,00
,00
2-AG (nM)
86
93
122
97
124
92
83
13
No pain (n=57)
Mild pain (n=28)
Moderate pain (n=23)
Severe pain (n=13)

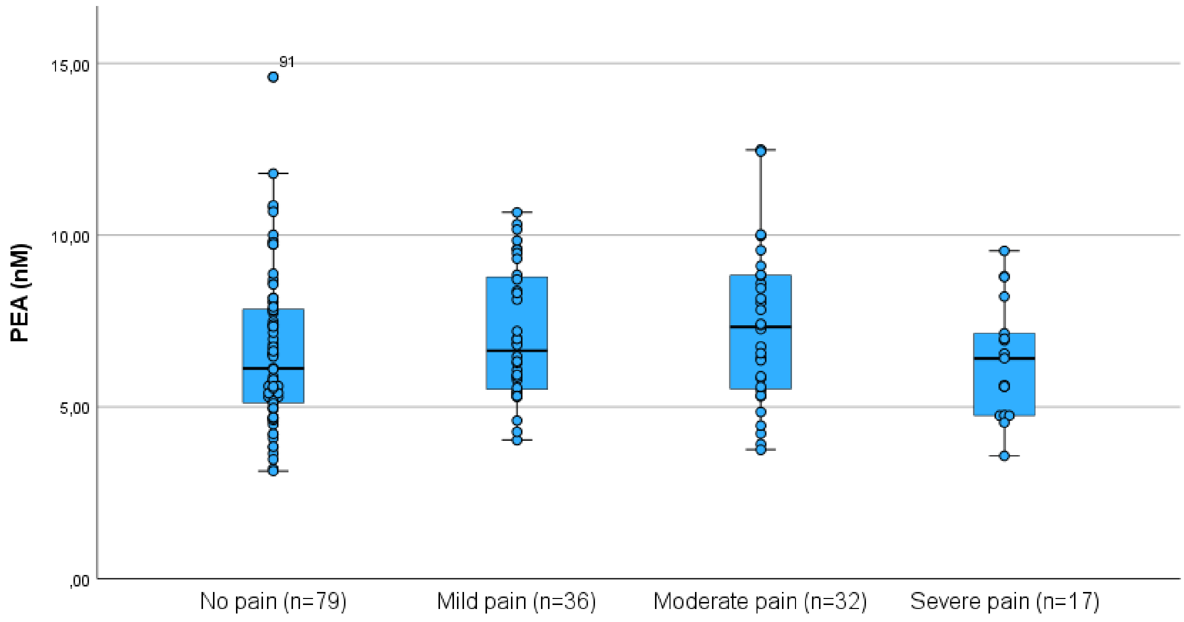
15,00
10,00
5,00
,00
PEA (nM)
91
No pain (n=79)
Mild pain (n=36)
Moderate pain (n=32)
Severe pain (n=17)

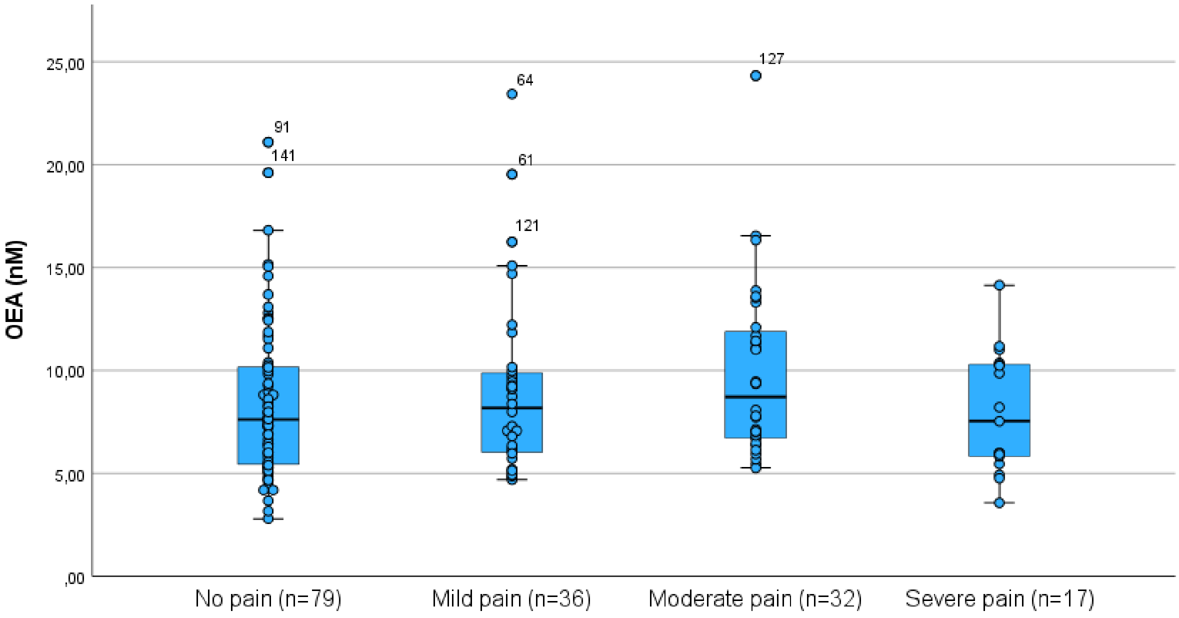
OEA (nM)
25,00
20,00
15,00
10,00
5,00
,00
91
141
64
61
121
127
No pain (n=79)
Mild pain (n=36)
Moderate pain (n=32)
Severe pain (n=17)

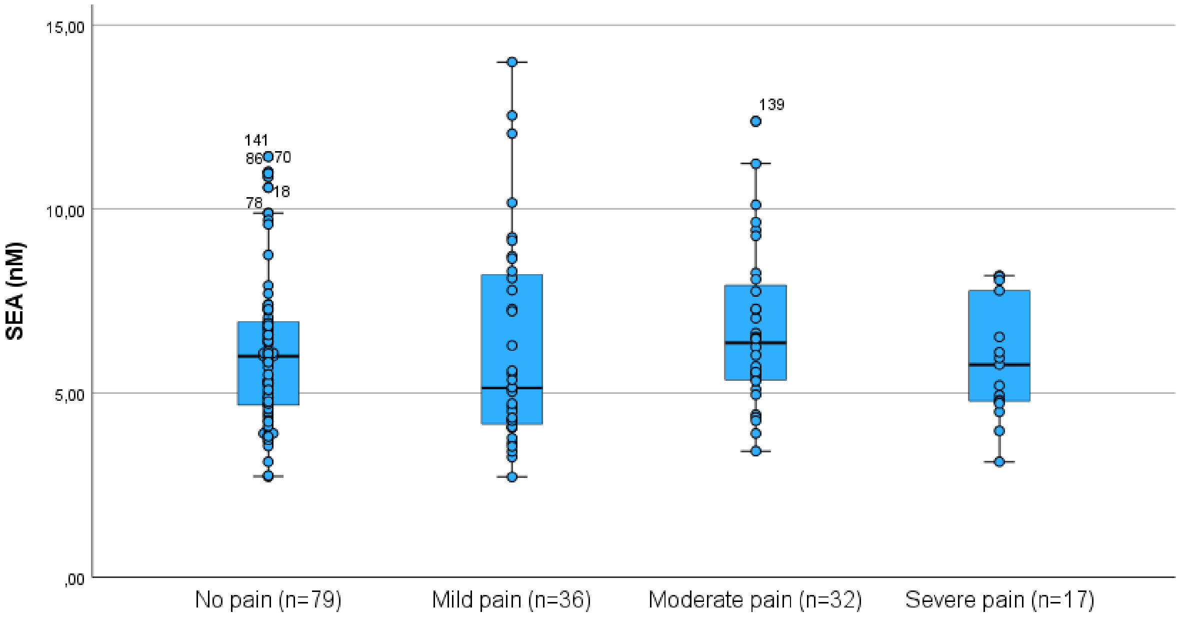
SEA (nM)
15,00
10,00
5,00
,00
141
86
70
18
78
139
No pain (n=79)
Mild pain (n=36)
Moderate pain (n=32)
Severe pain (n=17)

Online supplement 2

Correlation matrix between the 5 lipids with Spearman's rho and P-value (n=170).

		AEA	OEA	PEA	SEA	2-AG
**AEA**	Rho	**1**	**0.58***	**0.44***	**0.28***	**0.25***
	P-value		<0.001	<0.001	<0.001	0.007
**OEA**	Rho		**1**	**0.71***	**0.53***	**0.06**
	P-value			<0.001	<0.001	0.512
**PEA**	Rho			**1**	**0.52***	**0.13**
	P-value				<0.001	0.161
**SEA**	Rho				**1**	**0.23***
	P-value					0.01
**2-AG**	Rho					**1**
	P-value					

*Statistically significant at the 0.05 level; this remained significant at a false discovery rate of 20 %.
